# Anhysteretic Magneto-Elastic Behaviour of Terfenol-D: Experiments, Multiscale Modelling and Analytical Formulas

**DOI:** 10.3390/ma14185165

**Published:** 2021-09-08

**Authors:** Laurent Daniel, Mathieu Domenjoud

**Affiliations:** 1 Université Paris-Saclay, CentraleSupélec, CNRS, Laboratoire de Génie Electrique et Electronique de Paris, 91192 Gif-sur-Yvette, France; mathieu.domenjoud@centralesupelec.fr; 2 Sorbonne Université, CNRS, Laboratoire de Génie Electrique et Electronique de Paris, 75252 Paris, France

**Keywords:** magnetostriction, magnetisation, smart materials, multiaxial stress

## Abstract

Giant magnetostrictive materials such as Terfenol-D and Galfenol are used to design actuators and sensors, converting magnetic input into a mechanical response, or conversely, mechanical input into a magnetic signal. Under standard operating conditions, these materials are subjected to stress. It is therefore important to be able to measure, understand and describe their magneto-mechanical behaviour under stress. In this paper, a comprehensive characterisation of the anhysteretic magneto-mechanical behaviour of Terfenol-D was performed. An energy-based multiscale approach was applied to model this behaviour. Finally, it was shown that the strain behaviour of Terfenol-D can be satisfactorily described using an analytical model derived from the full multiscale approach.

## 1. Introduction

Magnetic and mechanical behaviours are strongly coupled in ferromagnetic materials. Magnetisation is sensitive to the application of stress, leading to significant effects on the performance of electromagnetic devices [1,2,3]. Conversely, the magnetisation process is associated with a mechanical deformation called magnetostriction [4]. Magnetostriction can notably be used for actuation purposes, for instance using giant magnetostrictive materials (GMM) [5,6,7,8]. Magnetostriction strain itself is very sensitive to the level of stress [9,10]. In this context, the modelling of magneto-elastic behaviour has attracted interest from many researchers. Magneto-elastic approaches have for instance been developed as extensions of Jiles-Atherton [11,12,13,14] or Preisach [15,16] models. Thermodynamic [17,18,19] and multiscale [20,21,22,23,24,25,26,27,28,29] approaches have been specifically developed to describe the combined effects of magnetic field and multiaxial stress on ferromagnetic materials. These multiscale approaches can be simplified [30,31,32,33] and under very strong assumptions, can provide analytical formulas for the description of magneto-elastic couplings [34,35,36]. This paper aims at demonstrating the capability of such analytical approaches to provide a good approximation of the strain behaviour of GMM. In a first part, the anhysteretic magneto-elastic behaviour of Terfenol-D is characterised. Particular attention is given to the experimental boundary conditions since a lack of control can lead to measurements errors of up to 40% on the longitudinal strain [37]. An energy-based multiscale approach [23] is then briefly presented and applied to Terfenol-D. The simplification into an analytical formula [34,35] is finally presented. The comparison with the experimental measurements allows validating the approach for 1D stress configurations, and the comparison with the full multiscale approach allows drawing conclusions for multiaxial stress cases.

## 2. Experimental Characterisation of Terfenol-D

### 2.1. Experimental Setup

**Figure 1:**
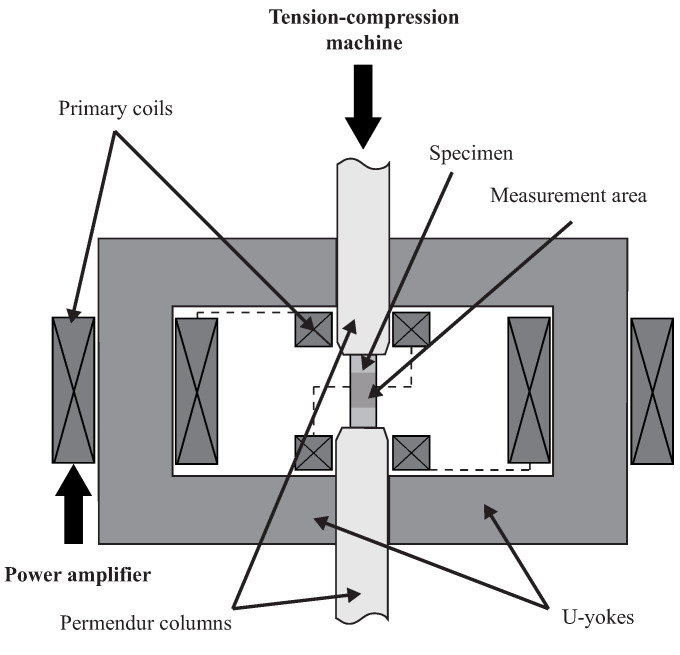
Magneto-mechanical characterisation device.

An experimental setup dedicated to the characterisation of GMM magneto-mechanical behaviour under constant stress [37] was used for this study. It consists of a magnetic circuit inserted into an electromechanical compression machine (Figure 1). All measurements were performed on cylindrical polycrystalline Terfenol-D rods (30 mm height, 5 mm radius) from TdVib LLC, USA. This configuration ensures that the demagnetisation fields are minimised and the form-effect is negligible [37]. The magnetic field was measured using a Hall probe, the magnetic induction through the integration of the induced voltage of a B-coil wound around the sample, and the strain through rosette strain gages glued on its surface. Specific attention was given to the control of the mechanical boundary conditions to ensure constant stress in the sample while it was subjected to dynamic magnetic loading. Indeed, it has been shown by Domenjoud et al. [37] that uncontrolled boundary conditions can be responsible for errors of several percent on the magnetic induction measurement, and up to 40% and 30% on the longitudinal and transverse strain measurements, respectively. Here, the variations in stress during magnetic loading were maintained below 0.1 MPa thanks to an active compensation of stress performed with a piezoelectric actuator.

### 2.2. Anhysteretic Measurements

While the results presented by Domenjoud et al. [37] were restricted to hysteresis loops, the original results shown here are dedicated to anhysteretic measurements. Such measurements allow isolating the reversible part of magneto-mechanical behaviour. To the best of our knowledge, there is no international standard for anhysteretic magnetic measurements—let alone for anhysteretic magneto-elastic measurements. Different procedures can be found in the literature (see for instance [38,39]). In this paper, each point of anhysteretic magnetisation and strain curves is acquired following the classical anhysteretic procedure presented by Jiles [40]. For a given point, after a major loop of amplitude $I_{\max}$ (in order to reach a magnetic state close to saturation), the material is subjected to a waveform $I_{\mathrm{anh}}$ which is an exponentially decaying sinusoidal current superimposed to a given bias current $I_{b}$. The input current is written as

(1) $$I_{\mathrm{anh}}\left(t\right)=I_{\max}\;\sin\left(2\pi ft\right)\;\exp\left(-kt\right)+I_{b}\;\left(1-\exp\left(-kt\right)\right).$$

**Figure 2:**
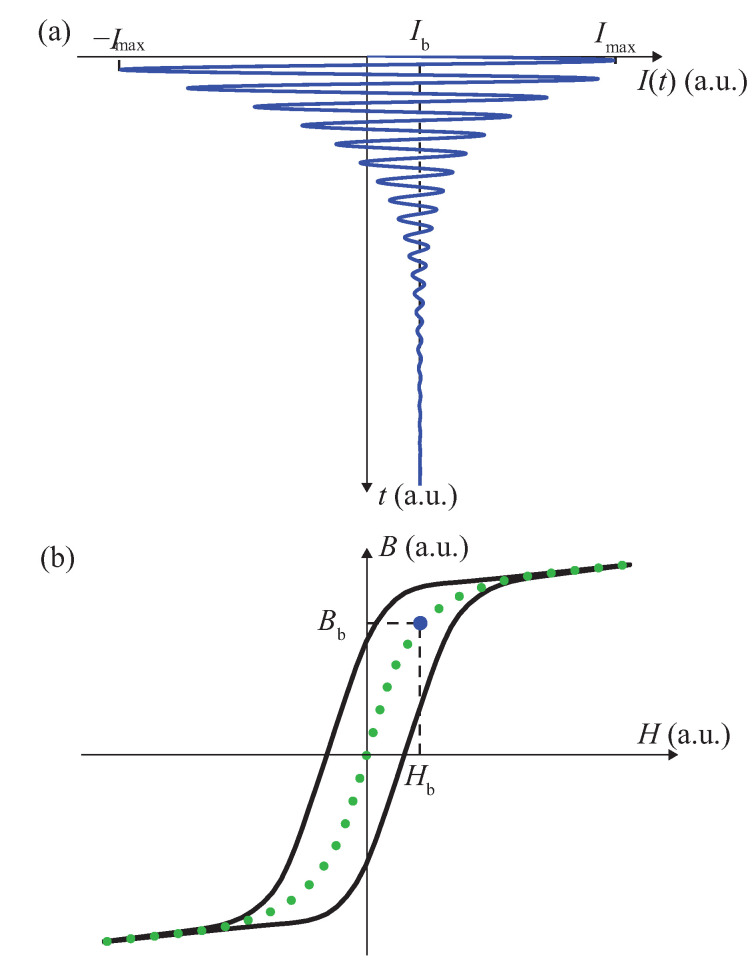
Magnetic excitation for the anhysteretic process (**a**), and the corresponding anhysteretic point (blue) of the complete anhysteretic curves (green) (**b**). This curve is included in the major magnetic loop. “a.u.” stands for “arbitrary units”.

The frequency *f* and damping parameter *k* in (1) are empirically set to be sufficiently small so that reducing them further would not change the measured value. For the results shown in this paper, the frequency *f* was set to 1 Hz and the damping parameter *k* to 0.34 s${}^{-1}$. The anhysteretic procedure is illustrated in Figure 2. Once the anhysteretic procedure is applied, a major loop (+$I_{\max}$ /$-I_{\max}$) is applied and the anhysteretic magnetic induction is calculated by comparison to the extremum (positive and negative) induction. Since the anhysteretic procedure can typically last up to 25 s, this final major loop allows avoiding the effects of a potential drift in the B-coil voltage. The strain was also measured during this loading pattern. Experiments were conducted on a single specimen, each anhysteretic point being collected only once. Repeatability aspects and the evaluation of measurement errors were investigated in a previous work [37]. The main source of error in the reproducibility of measurements comes from the precision in the application of stress. It is expected that this variability falls inside the error bar shown in the measurement results.

**Figure 3:**
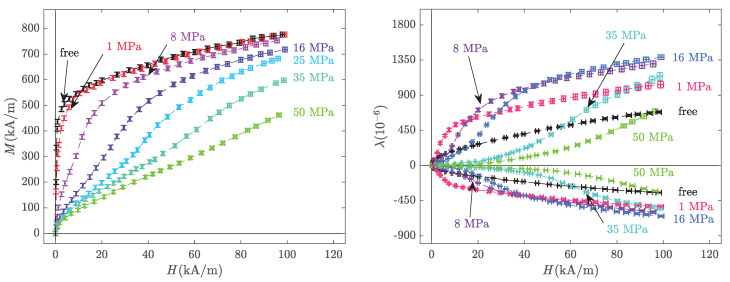
Anhysteretic magneto-elastic behaviour: magnetisation (**left**) and magnetostriction strain (**right**) at various levels of compressive stress with the corresponding error bars. Experimental results.

The anhysteretic procedure was successively applied to 25 different values of bias current $I_{b}$ and 22 levels of compressive stress from 0.025 to 90 MPa. The value 0.025 MPa, corresponds to the weight of the upper permendur column (see Figure 1). This stress state will be referred to as the stress-free state. Hence, a set of magnetisation and magnetostriction anhysteretic curves were obtained (Figure 3). At a given stress level, the reference strain state (zero strain) is defined as the deformation state of the demagnetised sample (under this given level of applied stress).

As already observed in previous works [6,9,41,42,43], the strong sensitivity of magnetisation and magnetostriction to pre-stress is evident in these measurements. For a given magnetic field, the magnetic induction decreases with applied stress. The effect of stress on magnetostriction is not monotonic. At a given magnetic field, the application of compressive stress first increases and then decreases the magnetostriction level. At the highest magnetic field, the maximum magnetostriction is observed for a stress of 16 MPa. As expected from the isochoric behaviour (no volume change) of an isotropic material, transverse magnetostriction is approximately half the longitudinal magnetostriction strain.

## 3. Multiscale Magneto-Elastic Model

### 3.1. Multiscale Modelling Principle

The multiscale approach for magneto-elastic behaviour [23,24,25] is based on an energy description of the equilibrium at the domain scale. A domain α is a region where the magnetisation $m_{\alpha }$ and magnetostriction strain $\varepsilon_{\alpha }^{\mu }$ are assumed to be uniform. They are defined as

(2) $$m_{\alpha }=M_{s}\;\alpha =M_{s}{\;}^{t}\left[\alpha_{1}\;\alpha_{2}\;\alpha_{3}\right]\;$$

(3) $$\mathrm{and}\;\varepsilon_{\alpha }^{\mu }=\frac{3}{2}\left(\begin{array}{ccc} \lambda_{100}\left(\alpha_{1}^{2}-\frac{1}{3}\right) & \lambda_{111}\alpha_{1}\alpha_{2} & \lambda_{111}\alpha_{1}\alpha_{3}\\ \lambda_{111}\alpha_{1}\alpha_{2} & \lambda_{100}\left(\alpha_{2}^{2}-\frac{1}{3}\right) & \lambda_{111}\alpha_{2}\alpha_{3}\\ \lambda_{111}\alpha_{1}\alpha_{3} & \lambda_{111}\alpha_{2}\alpha_{3} & \lambda_{100}\left(\alpha_{3}^{2}-\frac{1}{3}\right) \end{array}\right)\;,$$

where $M_{s}$ is the saturation magnetisation of the material, and $\left(\alpha_{1},\alpha_{2},\alpha_{3}\right)$ is the direction cosines of the magnetisation in the domain. $\lambda_{100}$ and $\lambda_{111}$ are the saturation magnetostriction constants of the crystal along directions the <100> and <111>, respectively, (assuming a cubic symmetry).

The free energy $W_{\alpha }$ of a magnetic domain α is decomposed as

(4) $$W_{\alpha }=W_{\alpha }^{mag}+W_{\alpha }^{an}+W_{\alpha }^{\sigma }+W_{\alpha }^{conf}\;$$

$W_{\alpha }^{mag}$ is the magnetostatic energy (5), which tends to favour domains with magnetisation $m_{\alpha }$ aligned with the applied magnetic field H. $\mu_{0}$ is the vacuum permeability:

(5) $$W_{\alpha }^{mag}=-\mu_{0}\;H.m_{\alpha }\;$$

$W_{\alpha }^{an}$ is the magneto-crystalline anisotropy energy, which tends to favour magnetisation $m_{\alpha }$ oriented along the easy axes. It is given by (6) in the case of a cubic symmetry. $K_{1}$ and $K_{2}$ denote the magnetocrystalline anisotropy constants of the material:

(6) $$W_{\alpha }^{an}=K_{1}\left(\alpha_{1}^{2}\alpha_{2}^{2}+\alpha_{2}^{2}\alpha_{3}^{2}+\alpha_{3}^{2}\alpha_{1}^{2}\right)+K_{2}\left(\alpha_{1}^{2}\alpha_{2}^{2}\alpha_{3}^{2}\right)\;$$

$W_{\alpha }^{\sigma }$ is the magneto-elastic energy, incorporating the effect of stress on the domain equilibrium. It is given by (7) where σ is the stress second-order tensor:

(7) $$W_{\alpha }^{\sigma }=-\sigma :\varepsilon_{\alpha }^{\mu }\;$$

$W_{\alpha }^{conf}$ is a complementary term that can be introduced to consider the possible bias in the initial domain configuration, created for instance by residual stresses or shape anisotropy [44]. This configuration term (8) was chosen here as the result of the effect of a (fictitious) uniaxial pre-stress $\sigma_{0}$. In the following, this uniaxial pre-stress will be applied along the magnetic field direction x, and $\sigma_{0}$ will be treated as a material parameter.

(8) $$W_{\alpha }^{conf}=\sigma_{0}{\;}^{t}x\cdot \varepsilon_{\alpha }^{\mu }\cdot x$$

For a given single crystal, the free energy $W_{\alpha }$ can be evaluated in any direction α. In practice, the icosphere discretisation of a unit sphere can be used [23,44]. Once the free energy $W_{\alpha }$ is known for all domain families α, the volume fractions $f_{\alpha }$ of domain families α are introduced as internal variables. These internal variables can be calculated according to an explicit Boltzmann-type relation [21,24,44]:

(9) $$f_{\alpha }=\frac{\exp\;(-A_{s}\;W_{\alpha })}{\sum_{\alpha }\exp\;\left(-A_{s}\;W_{\alpha }\right)}\;$$

where $A_{s}$ is a material parameter, proportional to the initial slope $\chi^{o}$ of the unstressed anhysteretic magnetisation curve [24]:

(10) $$A_{s}=\frac{3\chi^{o}}{\mu_{0}M_{s}^{2}}$$

From the magnetisation (2), the magnetostriction strain (3) and volume fraction (9) of each domain family α, the magnetisation and the magnetostriction strain at the single crystal scale are obtained through a volume average over the single crystal.

Since these materials are usually polycrystalline, the operation is repeated for different grain orientations, representative for the crystallographic texture of the material [25]. In the case of an isotropic material, as supposed here, the crystallographic orientations can be randomly chosen or according to a specific regular distribution orientation function. In this paper, the regular zoning of the crystallographic orientation space used by Daniel et al. [24] was employed. It contains 546 grain orientations. The magnetisation M and magnetostriction strain $\varepsilon^{\mu }$ of the material are finally obtained by a volume average over all the grain orientations:

(11) $$M\;=\;\langle m_{\alpha }\rangle$$

(12) $$\varepsilon^{\mu }\;=\;\langle \varepsilon_{\alpha }^{\mu }\rangle$$

It is possible to account for the heterogeneity of the stress and magnetic field within the material using appropriate localisation operators [24,25]. In this paper, we assumed that the magnetic field H and the stress σ were uniform within the material. Neglecting the fluctuations of magnetic field and stress is a classical simplification to multiscale approaches, which allows a significant reduction in computation time [44]. A part of grain-to-grain interactions are then ignored. Although the quantitative predictions can be altered by such an assumption, the main features of the model are maintained.

### 3.2. Multiscale Modelling Results

The material parameters for the single crystal were taken from the literature, similarly to those used by Daniel and Galopin [23]. The crystallographic texture was assumed to be isotropic (regular orientation distribution function [24] as explained just above). Only two material parameters must then be identified: $A_{s}$ for the definition of the volume fractions (9) and $\sigma_{0}$ for the initial configuration energy (8). As evident from (10), $A_{s}$ can be identified from the initial slope of the stress-free anhysteretic magnetisation curve. It was taken here as 5 × 10${}^{-4}$ m${}^{3}\cdot$J${}^{-1}$. $\sigma_{0}$ was chosen so as to approximately fit the saturation magnetostriction strain $\lambda_{s}$ for the unstressed material. The measured saturation strain ($\lambda_{s}\approx 600\times 10^{-6}$) is indeed lower than the value expected for an isotropic polycrystal under uniform stress (Reuss assumption [24]: $\lambda_{s}=2\;/\;5\lambda_{100}+3\;/\;5\lambda_{111}\approx 1020\times 10^{-6}$). The initial configuration term acts as a compensation for this difference. It was found that a fictitious pre-tensile stress of 1 MPa was enough to correct this initial configuration effect. All the material parameters are summarised in Table 1.

**Figure 4:**
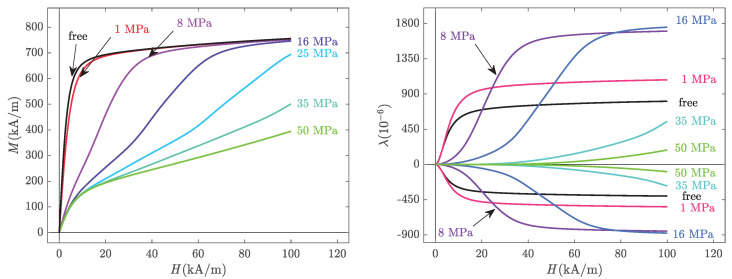
Anhysteretic magneto-elastic behaviour: magnetisation (**left**) and magnetostriction strain (**right**) at various levels of compressive stress. Modelling results from the full multiscale approach.

The modelling results are presented in Figure 4. It is recalled that except for the initial slope of the stress-free anhysteretic curve (to identify $A_{s}$) and the maximum stress-free magnetostriction strain (to identify $\sigma_{0}$), the magneto-mechanical measurements were not used to feed the modelling, so the results of Figure 4 should be considered as independent predictive results (rather than interpolation results).

The multiscale modelling results clearly describe the trends observed in the experiments (Figure 3). The magnetisation reaches saturation quicker compared to the experiments, but the effect of stress on the magnetisation curves is accurately predicted. The magnetostriction strain is slightly overestimated, but the effect of stress—and notably the various crossings of the different curves—is satisfactorily described. A better adjustment between the experiments and multiscale modelling could be obtained using a numerical fitting procedure of the measured data for the identification of the material parameters. The magnetostriction constants, for instance, may be overestimated in the modelling, which can explain some discrepancies. However, the approach of independently taking the material parameters from the literature was preferred as it demonstrates the predictive ability of the multiscale approach.

## 4. Analytical Magneto-Elastic Model

### 4.1. Analytical Constitutive Equations

From the full multiscale approach, and to the price of additional simplifying assumptions, it is possible to derive analytical formulas for the anhysteretic magnetisation M [34] and magnetostriction strain $\varepsilon^{\mu }$ [35] of homogeneous and isotropic ferromagnetic materials as functions of the applied magnetic field H and stress σ. The magnetisation is given by (13), where x is the orientation of the magnetic field, and *H* its norm (H=Hx):

(13) $$M=\frac{A_{x}^{m}\sinh\left(\kappa_{m}H\right)}{A_{x}^{m}\cosh\left(\kappa_{m}H\right)+A_{y}^{m}+A_{z}^{m}}\;M_{s}\;x$$

$A_{x}^{m}$ (resp. $A_{y}^{m}$, $A_{z}^{m}$) is a function of the applied mechanical stress σ (second order tensor):

(14) $$A_{x}^{m}=\exp\left(\alpha_{m}\;\sigma_{xx}\right)\;\left(resp.\sigma_{yy},\sigma_{zz}\right)$$

This expression for the magnetisation relies only on three material parameters ($M_{s}$, $\kappa_{m}$, $\alpha_{m}$). $\kappa_{m}$ is related to the material susceptibility (15). $\alpha_{m}$ describes the effect of stress (16). $\lambda_{s}$ is the saturation magnetostriction constant of the material. It is recalled that $M_{s}$ is the saturation magnetisation of the material and $\chi^{o}$ is the initial susceptibility of the anhysteretic magnetisation curve under no applied stress.

(15) $$\kappa_{m}=\frac{3\chi^{o}}{M_{s}}$$

(16) $$\alpha_{m}=\frac{9\lambda_{s}\chi^{o}}{2\mu_{0}M_{s}^{2}}$$

The magnetostriction tensor, in the isotropic and isochoric case, can be written in the form:

(17) $$\varepsilon^{\mu }=\lambda \left(\begin{array}{ccc} 1 & 0 & 0\\ 0 & -1\;/\;2 & 0\\ 0 & 0 & -1\;/\;2 \end{array}\right)$$

where λ is the longitudinal magnetostriction strain (parallel to the magnetic field H). It is given by (18). The expression also relies on three material parameters ($\lambda_{s}$, $\kappa_{\mu }$, $\alpha_{\mu }$).

(18) $$\lambda =\lambda_{s}\;\left(1-\frac{3(A_{y}^{\mu }+A_{z}^{\mu })}{2(A_{x}^{\mu }\cosh\left(\kappa_{\mu }H\right)+A_{y}^{\mu }+A_{z}^{\mu })}\right)$$

with

(19) $$A_{x}^{\mu }=\exp\left(\alpha_{\mu }\;\sigma_{xx}\right)\;\left(\mathrm{resp}.\sigma_{yy},\sigma_{zz}\right)$$

The original development of the model implies that $\kappa_{m}=\kappa_{\mu }$ and $\alpha_{m}=\alpha_{\mu }$. The magneto-elastic behaviour is then described by (13) and (18) using only three material parameters. In order to obtain a better fitting with experimental data, and compensate for the numerous simplifying assumptions, it may be convenient in practical applications to consider six independent parameters ($M_{s}$, $\kappa_{m}$, $\alpha_{m}$, $\lambda_{s}$, $\kappa_{\mu }$, $\alpha_{\mu }$). This option will be used in the following.

### 4.2. Analytical Modelling Results

The material parameters of the analytical model were identified using a limited number of experimental curves (two magnetisation curves and two magnetostriction curves), the rest of them being reserved for validation purposes. $M_{s}$ is identified as the maximum level of magnetisation under no applied stress. $\lambda_{s}$ is identified as the maximum level of magnetostriction strain under no applied stress. $\kappa_{m}$ and $\kappa_{\mu }$ are identified to best fit the magnetisation and magnetostriction curves (respectively) under no applied stress. Indeed, when no stress is applied, the parameters $\alpha_{m}$ and $\alpha_{\mu }$ vanish from (13) and (18). Finally, $\alpha_{m}$ and $\alpha_{\mu }$ were identified as the best fit to the magnetisation and magnetostriction curves (respectively) under 16 MPa applied stress. The obtained material parameters are given in Table 2.

It can be noticed that $\kappa_{m}$ and $\kappa_{\mu }$ are reasonably close to each other, and that $\alpha_{m}$ and $\alpha_{\mu }$ are equal. This was expected, as discussed above. The use of four independent parameters would probably be sufficient to describe the material behaviour. The use of six different parameters only allows a better fitting of the experimental data. It can also be noticed that the identified value for $M_{s}$ ($7.8\times 10^{5}$ A/m) is slightly different from the physical material parameter ($8.0\times 10^{5}$ A/m, see Table 1). This results from the identification procedure from experimental measurements, which implies deviation from independently measured intrinsic material parameters.

**Figure 5:**
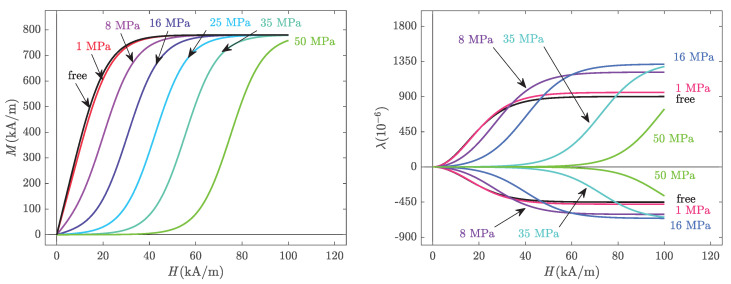
Anhysteretic magneto-elastic behaviour: magnetisation (**left**) and magnetostriction strain (**right**) at various levels of compressive stress. Modelling results from analytical modelling.

Once the material parameters of Table 2 were identified from two magnetisation and two magnetostriction curves, the analytical model can be validated using the remaining twenty magnetisation and twenty magnetostriction curves. For the sake of clarity, however, only part of the data were selected in the following figures, as for the previous ones. Figure 5 shows the magnetisation and magnetostriction curves of Terfenol-D obtained from the analytical Formulas (13) and (18), respectively.

The model has a general tendency to overestimate the magnetic induction. This is due to the fact that the magnetisation process shows two stages: a first one dominated by domain wall motion and a second one dominated by magnetisation rotation. The simplified analytical modelling does not consider these two stages and the magnetisation curves tend to be much steeper to reach saturation compared to the experimental results. It can also be noticed that for low fields, the effect of stress is largely overestimated, leading to very low magnetic permeabilities, whereas the experimental results show that this strong degradation is more gradual and appears for intermediate field levels, from a few kA/m. The effect of stress is only qualitatively described.

**Figure 6:**
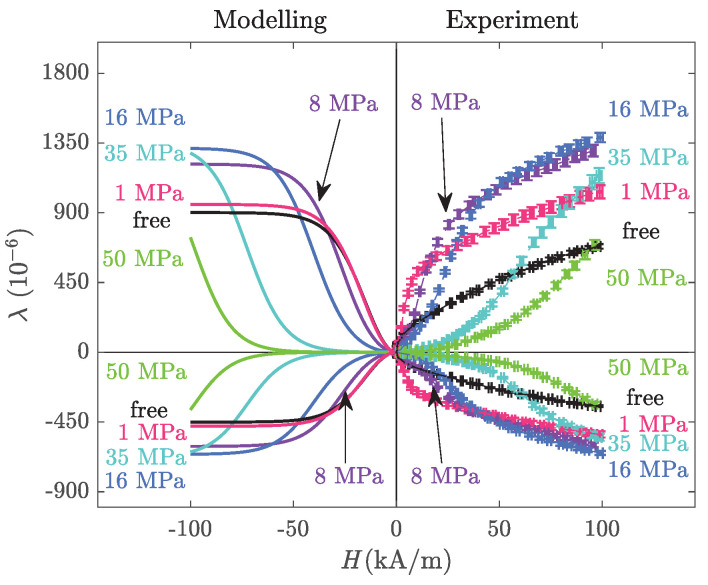
Longitudinal (**top**) and transverse (**bottom**) anhysteretic magnetostriction strain under uniaxial compressive stress: analytical model (**left**) and experimental results with the corresponding error bars (**right**).

On the contrary, the magnetostriction strain is well described by the analytical modelling. This is evident in Figure 6 showing the comparison between analytical modelling and experimental results for the longitudinal and transverse magnetostriction curves.

It is noticeable in the longitudinal strain evolution that the non-linearity of the effect of stress on the maximum level of magnetostriction is captured by the analytical model. Stress (up to 16 MPa) first increases the maximum level of magnetostriction, and then decreases it. This is due to the ΔE effect [45]. Indeed, although the curves were arbitrarily shifted so as to start from zero strain at zero magnetic field, there is an initial magnetostriction strain due to stress. As explained in detail in [35], the analytical model captures this so-called ΔE effect. The same conclusions can be drawn from the transverse strain analysis: since the behaviour of Terfenol-D is isotropic, the definition that the transverse strain is half the longitudinal one with opposite sign is well verified.

## 5. Modelling 3D Configurations

Both multiscale and analytical approaches are intrinsically multiaxial. They can naturally consider 3D stress configurations. On the one hand, the multiscale model is based on the physical mechanisms responsible for magneto-elasticity. It relies on standard physical parameters usually available in the literature and on only two adjustment parameters identified from stress-free measurements. Its predictive ability is strong. On the other hand, the analytical model is a very simple formula. The material parameters can be obtained by fitting a limited number of experimental measurements. Its implementation is straightforward, but due to the numerous assumptions made, its predictive ability can be questioned.

**Figure 7:**
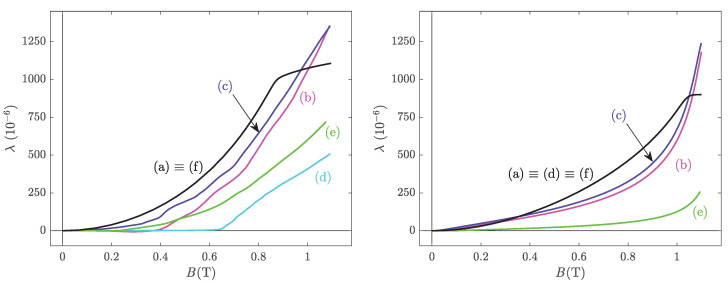
Predicted longitudinal anhysteretic magnetostriction strain under stress-free (a); 1D compression (b); equibiaxial compression (c); pure shear at 0${}^{\circ }$ with respect to the magnetisation (d); pure shear at 45${}^{\circ }$ with respect to the magnetisation (e); and hydrostatic pressure (f). The stress amplitude σ is set at −40 MPa.

In this section, the predictive ability of the analytical model is evaluated by comparison to the full multiscale approach in typical 3D configurations. Figure 7 shows the predicted longitudinal magnetostriction strain as a function of the magnetic induction, under several stress conditions: stress-free (a); 1D compression (b); equibiaxial compression (c); pure shear at 0${}^{\circ }$ with respect to the magnetisation (d); pure shear at 45${}^{\circ }$ with respect to the magnetisation (e); and hydrostatic pressure (f). The corresponding stress tensors are given, respectively, by

$$\begin{array}{ccc} \sigma^{a}=\left(\begin{array}{ccc} 0 & 0 & 0\\ 0 & 0 & 0\\ 0 & 0 & 0 \end{array}\right) & \sigma^{b}=\left(\begin{array}{ccc} \sigma & 0 & 0\\ 0 & 0 & 0\\ 0 & 0 & 0 \end{array}\right) & \sigma^{c}=\left(\begin{array}{ccc} \sigma & 0 & 0\\ 0 & \sigma & 0\\ 0 & 0 & 0 \end{array}\right)\\ \sigma^{d}=\left(\begin{array}{ccc} 0 & \sigma & 0\\ \sigma & 0 & 0\\ 0 & 0 & 0 \end{array}\right) & \sigma^{e}=\left(\begin{array}{ccc} \sigma & 0 & 0\\ 0 & -\sigma & 0\\ 0 & 0 & 0 \end{array}\right) & \sigma^{f}=\left(\begin{array}{ccc} \sigma & 0 & 0\\ 0 & \sigma & 0\\ 0 & 0 & \sigma \end{array}\right) \end{array}.$$

The compression stress amplitude is arbitrarily set to 40 MPa. The left and right pictures of Figure 7 show the results for the full multiscale approach and for the analytical model, respectively.

It can be seen, as expected, that the form of the stress tensor strongly modifies the effect on the magnetostriction strain. All the considered stress configurations tend to deteriorate the magnetostrictive response, at least below 1 T. As expected, magnetostriction is not sensitive to hydrostatic pressure (both models). The analytical model is close to the multiscale model in cases (a), (b), (c) and (f). This means that under uniaxial, equibiaxial and hydrostatic configurations, the analytical model can be used with reasonable confidence. On the contrary, the discrepancy is strong in cases (d) and (e). This means that the shear configurations are not handled well by the analytical approach. It is particularly remarkable that the analytical formula is insensitive to pure shear when applied at 0${}^{\circ }$ with respect to the magnetic field direction (see (18)). This is a known drawback of the simplified multiscale approach [46]. It can also be noticed—both from experimental and modelling points of view—that in contrast to some standard assumption, magnetostriction cannot be accurately described as a quadratic stress-independent function of the magnetic induction.

## 6. Conclusions

This work presents a study on the anhysteretic magneto-elastic behaviour of Terfenol-D and on the ability of 3D modelling approaches to capture the complexity of this behaviour. A full experimental campaign was first presented, including magneto-elastic measurements at different levels of constant compressive stress. A multiscale modelling approach was then applied to the material, and the predictivity of the model was shown to be very satisfactory. Two very simple analytical formulas were then proposed to describe the effects of stress on both the anhysteretic magnetisation and magnetostriction of ferromagnetic materials. These two formulas require six independent material parameters. The number of parameters can be reduced down to three if required, although it would deteriorate the accuracy of the description of magneto-elastic effects. It was shown that the description of the effect of stress on magnetisation curves is only qualitative. A strong overestimation of the effect of stress limits the performance of the model. On the contrary, it was shown that the main features of the effect of stress on the magnetostriction strain are well captured by the approach. The analytical formula provides consistent results under uniaxial, biaxial and triaxial configurations by comparison to the multiscale model. Shear stress configurations, however, were shown to be a weakness of the analytical approach. It is worth noting that the two approaches discussed in this paper naturally incorporate three-dimensional stress tensors. In the cases where the analytical model showed satisfying results, the formulas provide a very convenient tool for engineers. They are very easy to implement into numerical software for structural analysis and can be used as a pre-design tool for electromagnetic devices before more comprehensive approaches are used for validation.

## Figures and Tables

**Table 1:** Modelling parameters for the multiscale model.

Parameter	Value	Unit	Source
$M_{s}$	8 × 10${}^{5}$	A·m${}^{-1}$	Sandlund et al. [41]
$\left(K_{1},K_{2}\right)$	(−0.8, −1.8) × 10${}^{5}$	J·m${}^{-3}$	Engdahl [6]
$\left(\lambda_{100},\lambda_{111}\right)$	(9, 164) × 10${}^{-5}$	-	Jiles [40]
$A_{s}$	5 × 10${}^{-4}$	m${}^{3}\cdot$J${}^{-1}$	-
$\sigma_{0}$	1	MPa	-

**Table 2:** Modelling parameters for the analytical model.

Parameter	Value	Unit	Parameter	Value	Unit
$M_{s}$	$7.8\times 10^{5}$	A/m	$\lambda_{s}$	$9.0\times 10^{-4}$	-
$\kappa_{m}$	$1.4\times 10^{-4}$	m/A	$\kappa_{\mu }$	$1.1\times 10^{-4}$	m/A
$\alpha_{m}$	$1.9\times 10^{-7}$	Pa${}^{-1}$	$\alpha_{\mu }$	$1.9\times 10^{-7}$	Pa${}^{-1}$
